# Ex Vivo Vibration Spectroscopic Analysis of Colorectal Polyps for the Early Diagnosis of Colorectal Carcinoma

**DOI:** 10.3390/diagnostics11112048

**Published:** 2021-11-04

**Authors:** Alla Synytsya, Aneta Vaňková, Michaela Miškovičová, Jaromír Petrtýl, Luboš Petruželka

**Affiliations:** 1 Department of Analytical Chemistry, University of Chemistry and Technology Prague, Technická 5, 166 28 Prague, Czech Republic; vankovaa@vscht.cz; 2 Department of Oncology, First Faculty of Medicine, Charles University in Prague and General University Hospital in Prague, U Nemocnice 2, 128 00 Prague, Czech Republic; michaela.miskovicova@vfn.cz (M.M.); lubos.petruzelka@vfn.cz (L.P.); 3 4th Internal Clinic—Gastroenterology and Hepatology, First Faculty of Medicine, Charles University in Prague and General University Hospital in Prague, U Nemocnice 2, 128 00 Prague, Czech Republic; jaromir.petrtyl@vfn.cz

**Keywords:** colon polyps, colorectal carcinoma, early cancer diagnosis, vibrational spectroscopy, chemometrics

## Abstract

Colorectal cancer is one of the most common and often fatal cancers in humans, but it has the highest chance of a cure if detected at an early precancerous stage. Carcinogenesis in the colon begins as an uncontrolled growth forming polyps. Some of these polyps can finally be converted to colon cancer. Early diagnosis of adenomatous polyps is the main approach for screening and preventing colorectal cancer, and vibration spectroscopy can be used for this purpose. This work is focused on evaluating FTIR and Raman spectroscopy as a tool in the ex vivo analysis of colorectal polyps, which could be important for the early diagnosis of colorectal carcinoma. Multivariate analyses (PCA and LDA) were used to assist the spectroscopic discrimination of normal colon tissue, as well as benign and malignant colon polyps. The spectra demonstrated evident differences in the characteristic bands of the main tissue constituents, i.e., proteins, nucleic acids, lipids, polysaccharides, etc. Suitable models for discriminating the three mentioned diagnostic groups were proposed based on multivariate analyses of the spectroscopic data. LDA classification was especially successful in the case of a combined set of 55 variables from the FTIR, FT Raman and dispersion Raman spectra. This model can be proposed for ex vivo colorectal cancer diagnostics in combination with the colonoscopic extraction of colon polyps for further testing. This pilot study is a precursor for the further evaluation of the diagnostic potential for the simultaneous in vivo application of colonoscopic Raman probes.

## 1. Introduction

Colorectal cancer is one of the most common and often fatal cancers in humans globally. However, among all malignant neoplasms of the intestine, it has the highest chance of a cure if it is detected at an early stage as a precancerous lesion [1]. Carcinogenesis in the colon or rectum begins as uncontrolled growth in the inner layers towards the intestinal lumen, forming polyps. Some of these polyps, known as neoplastic or adenomatous, can undergo a gradual transformation, and most of them can finally be converted to colon cancer [2,3]. This gradual development of normal intestinal epithelium into carcinoma occurs as a result of several successive genetic mutations, some of which are inherited, while others are acquired [4]. These changes usually occur slowly over many years, so the disease can be prevented by early detection and through the removal of adenomas before they develop into cancer.

Early diagnosis and endoscopic resection of adenomatous polyps is the main approach for the screening and prevention of colorectal cancer. Colonoscopy and histopathology are now the standard methods for screening and diagnosing colorectal tissues. Colonoscopy in combination with polypectomy is able to reduce the incidence and mortality of colorectal cancer [5]. Colonoscopy is currently the most accurate and versatile diagnostic test for colorectal carcinoma because it can localize and biopsy lesions throughout the colon, detect neoplasms, and remove adenomatous polyps that could be precursors of cancer. Although colonoscopic screenings have significantly increased the survival rate of patients with colorectal cancer, it remains a challenge to distinguish adenomas and early adenocarcinomas from benign hyperplastic polyps using colonoscopy. This difficulty is mainly because conventional white light reflection colonoscopy relies heavily on the subjective visual assessment of colorectal polyps.

To make a definitive diagnosis of colorectal carcinoma, samples of suspicious tissue biopsies should be subjected to histological examination based on microscopic examination using paraffinization and specific dyes. A waxed tissue block is cut into very thin slices. After further treatments, the slices are placed onto a sample slide and examined under a microscope to determine whether it is a tumor, benign or malignant, and what type it belongs to [6,7,8]. However, routine histopathological methods applied to analyses of suspected cancerous or precancerous lesions have several disadvantages. Sample preparation procedures with an evaluation of the tissue last approximately 7–10 days, and the entire result often depends on the experience of pathologists and can be highly subjective. Therefore, despite the common diagnostic methods mentioned above, there is still a request for novel, effective, and non-expensive approaches for the early diagnosis of adenomatous polyps for the prevention of colorectal cancer.

Vibration spectroscopy, both infrared and Raman, can be a powerful tool for the early diagnosis of colorectal cancer in several main areas: (i) ex vivo analysis of biopsies of colorectal tissue, including polyps removed by colonoscopy; (ii) endoscopic analysis of the colon cavity in vivo; (iii) analysis of intact intestinal epithelial cells; (iv) visualization and mapping of histological sections of colorectal tissues, and (v) analysis of the biological fluids and derived samples (blood plasma, isolated serum proteins, etc.). It has been shown that both of these methods can support the standard techniques mentioned above and can significantly improve the clinical diagnosis of colon cancer [9,10,11]. Various methods and techniques of vibrational spectroscopy have proven to be effective in the analysis of colorectal tissues in vivo [12,13,14], in situ or ex vivo [15,16,17,18]. Single living cells of the intestinal epithelium will also be subjected to vibrational spectroscopic evaluation in order to diagnose colorectal cancer [19,20,21]. Distinguishing between benign and malignant polyps can be achieved via spectroscopic visualization of their histological characteristics, including tissue [7,22,23] and subcellular [24] structures. Moreover, non-invasive analysis of the blood serum [15,25], blood plasma [26,27], or isolated serum proteins [28,29] by chiroptical and vibrational spectroscopy could also help in the early detection of this disease.

To distinguish between normal, benign, and malignant colon tissue samples based on collected spectroscopic data, it is often necessary to employ multivariate statistical methods to extract diagnostic information that is not available in conventional spectral comparisons. Statistical methods used for spectroscopic diagnostics can be divided into controlled and uncontrolled methods [30]. To make a decision, the former approach relies only on vibrational spectra, while the latter relies on additional information obtained by the gold standard method, which is most often from classical histopathology. Principal component analysis (PCA) is often used as an unsupervised approach [30,31,32,33]. This technique reduces the number of variables and evaluates the spectroscopic data in the first approximation. A controlled approach is then introduced, such as linear discriminant analysis (LDA), which takes advantage of PCA and the histopathological findings to help classify tissue samples more efficiently [30,31,34,35]. For example, infrared spectral histopathology is able to distinguish between different types of tissues and their pathologies in sections of colon cancer tissue [7]. A supervised algorithm based on the random forest methodology was trained using classical histopathology, and was then used to identify tissue types and areas of colon adenocarcinoma. A high correspondence was shown between the images obtained by immunohistochemistry and classical and infrared spectral histopathology, with the latter method being able to detect changes in the composition of intestinal tissue without the need for labeling.

The aim of this work is an evaluation of vibrational spectroscopic methods, i.e., Fourier transform (FT) infrared spectroscopy (FTIR) with attenuated total reflection (ATR), FT Raman (λ_ex_ = 1064 nm), and dispersion Raman (λ_ex_ = 785 nm) spectroscopy as tools in the ex vivo analysis of colorectal polyps for the identification stage development of adenomatous polyps, which might be important for the early diagnosis of colorectal carcinoma. Multivariate analyses, namely PCA and LDA, were used to assist in the discrimination of normal, benign, and malignant colon tissues via vibrational spectroscopy.

## 2. Materials and Methods

### 2.1. Human Colorectal Tissue Samples

Ten patients aged 52 to 85 years undergoing routine colonoscopic examination at the 4th Internal Clinic—Gastroenterology and Hepatology of the 1st Faculty of Medicine and the General University Hospital in Prague (Charles University, Prague, Czech Republic) participated in this study. Colorectal polyps measuring 3–6 mm were taken with disposable biopsy forceps, and were subjected to a histological examination and spectroscopic analyses. Seven of the participating patients had histologically proven adenomatous colon polyps of low grade G1, and three patients had adenocarcinomatous polyps of intermediate grade G2: (i) polyp of invasive moderately differentiated adenocarcinoma of ascending colon with extensive involvement, (ii) polyp of invasive colon mucosal carcinoma with submucosal involvement, and (iii) polyp with structural features of invasive rectal adenocarcinoma. As samples of normal colon tissue, histologically confirmed samples of the non-tumor area of the colon were used, obtained during elective surgery for the medical treatment of colorectal cancer in fourteen patients of the Department of Oncology of the 1st Faculty of Medicine, Charles University and General University Hospital in Prague. All patients who kindly agreed to participate in this project signed their informed consent, and ethical approval for this study was obtained from the Ethics Committee of the 1st Faculty of Medicine and the General University Hospital in Prague.

### 2.2. Spectroscopic Measurements

Colon tissue samples measuring 3–6 mm were gently washed in 0.9% NaCl and were quickly dried on filter paper to remove the excess wash solution. Then, the samples were placed on a sample slide and analyzed via FTIR and Raman spectroscopy at six independent sites at 22 °C.

The FTIR spectra of the colon tissue samples were recorded on a Nicolet iS50 FTIR spectrometer (Thermo Fisher Scientific, Waltham, MA, USA) using the ATR accessory (ZnSe crystal) with a resolution of 4 cm^−1^ in the range of 4000–400 cm^−1^. The samples were dried on filter paper, placed on an ATR crystal, and then evenly pressed by a calibrated pressure tower. Five hundred and twelve scans were collected for each spectrum. Smoothing, ATR and baseline corrections were performed using the OMNIC 8.2 software (Thermo Fisher Scientific, Waltham, MA, USA).

The FT Raman spectra of colon tissue samples were recorded on a Bruker FT Raman (FRA 106/S, Equinox 55/S) spectrometer (Bruker, Billerica, MA, USA) equipped with a quartz beam splitter and a Ge detector cooled with liquid N_2_. A Nd:YAG laser (λ_ex_ = 1064 nm, Coherent, Santa Clara, CA, USA) was used for excitation. The power of the laser focused on the sample was ~60 mW. One thousand and twenty four independent scans were co-added to generate each individual Raman spectrum with a resolution of 2 cm^−1^.

Dispersion Raman spectra (region 400–3100 cm^−1^, resolution 2 cm^−1^) of the colon tissue samples were recorded on a B&W Tek i-Raman Plus spectrometer (B&W Tek Inc., Newark, DE, USA) equipped with a diode laser (λ_ex_ = 785 nm, power 90 mW, which corresponds to 20% of the maximal power 450 mW) and a BAC 151A Raman microscope with a 100× objective. These objectives are capable of providing a laser spot diameter of approximately 10–12 μm. Each spectrum was generated by the accumulation of 20 scans with a laser exposure time of 20 s per scan. No spectral or visual changes were observed during the scanning procedure.

The model reference compounds (pork fat, phosphatidylcholine from egg yolk, calf thymus DNA, myoglobin from equine skeletal muscle, human serum albumin (HSA), collagen from rat tail tendon, sodium hyaluronate from rooster comb, chondroitin sulfate A sodium salt from bovine trachea, and glycogen from bovine liver) corresponding to major tissue macromolecules were purchased from Sigma Aldrich (St. Louis, MO, USA). The spectroscopic measurements of these compounds were made under the same conditions as the measurements of the colon tissue samples.

### 2.3. Statistical Analyses of Spectroscopic Data

Vibrational spectra of the colon tissue samples recorded at independent sites were exported to Origin 6.0 (Microcal Origin, Northampton, MA, USA) software as the ASCII data files for further processing (smoothing, baseline correction, etc.). The spectra were normalized according to the appropriate tissue marker bands, i.e., amide vibrations in proteins ~1650 cm^−1^ and ~3290 cm^−1^ for FTIR and CH stretching vibrations at ~2930 cm^−1^ for FT Raman and dispersion Raman. Then the normalized spectra were imported to Unscrambler X 10.5.1 (CAMO Software AS, Oslo, Norway) software for a statistical evaluation, using PCA to reduce the highly correlated multidimensional spectroscopic datasets to a smaller number of uncorrelated principal components (PCs). The results of the PCA were represented by loading and component score plots. Comparing the PCA loading curves of the three chosen PCs, three sets of 15–20 variables in each for FTIR (20), FT Raman (15), and dispersion Raman (20) data were selected, normalized, and applied for LDA classification using XLSTAT (Addinsoft, Paris, France) software. The quality of these models was evaluated by the leave-one-out cross-validation procedure. The LDA results were represented by the squared Mahalanobis distances, variables/factor correlation, and factor score plots. The distances between the three diagnostic groups (normal colon tissues, benign and malignant colon polyps) were calculated from the squared Mahalanobis distances according to Equation (A1) (see Appendix A).

## 3. Results

### 3.1. Vibrational Spectra of Normal Colon Tissues, and Adenomatous and Adenocarcinomatous Polyps

**Figure 1:**
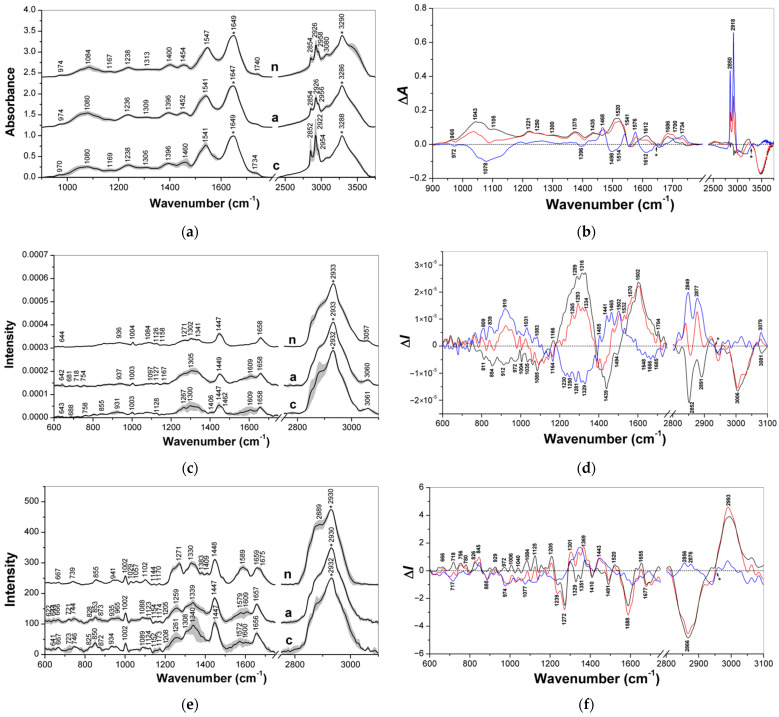
(Left panels) The average (black solid lines) and standard deviation (SD; gray space) FTIR (**a**), FT Raman (**c**) and dispersion Raman (**e**) spectra of the normal colon tissues “n”, and adenomatous “a” and adenocarcinomatous “c” colon polyps. (Right panels) The FTIR (**b**), FT Raman (**d**), and dispersion Raman (**f**) difference spectra adenoma minus normal (“a–n”, black), carcinoma minus normal (“c–n”, red) and carcinoma minus adenoma (“c–a”, blue); the average spectra were normalized at wavenumbers with an asterisk.

Average (mean ± SD) vibrational spectra of control colon tissues (*n* = 10), adenomatous (*n* = 7) and adenocarcinomatous (*n* = 3) colon polyps and the corresponding difference spectra (adenoma minus normal, carcinoma minus normal and carcinoma minus adenoma) are represented in Figure 1 left and right panels, respectively; the FTIR (900–3750 cm^−1^), FT Raman (500–3100 cm^−1^) and dispersion Raman (400–3100 cm^−1^) spectra are grouped in the top, middle and bottom panels, respectively. The band assignments are summarized in Table S1 in accordance with literature [17,36,37,38,39,40,41,42,43,44,45,46,47,48,49,50] and the corresponding vibration spectra of the model reference compounds (Figure S1a–c).

The analysis of the vibrational spectra is sometimes complicated, since the absorption bands often overlap with one another. Second derivative spectroscopy is a technique that helps detect overlapping bands. Therefore, the positions of the shoulders in the spectra were determined using the second derivative algorithm.

#### 3.1.1. FTIR Spectra (900–3750 cm^−1^)

As shown in Figure 1a, the characteristic bands of the proteins at 3286–3290 cm^−1^ (amide A), 1647–1649 cm^−1^ (amide I) and 1541–1547 cm^−1^ (amide II) were predominate in the FTIR spectra of all of the samples [36,38,39]. The amide II band of adenomatous and adenocarcinomatous polyps was shifted by 6 cm^−1^ to lower wavenumbers compared with the control colon tissue. A more detailed analysis of the spectral region of this band was made using the second derivatives, which showed two minima at 1547 and 1541 cm^−1^ (Figure S2). In the raw normal colon tissues—adenomatous polyps—adenocarcinomatous polyps, the first component decreased while the second component grew. Similar changes were observed in the region of the smaller negative band at 1576 cm^−1^ (C=C stretching vibration in aromatic amino acids or nucleic bases), which corresponded to the shoulder in the adenocarcinoma spectrum. The perturbations in the region of amide II could be associated with a change in the composition of proteins, as the position of this band varied greatly in different proteins (Figure S1a). The FTIR spectra also contained several bands at 2954–2958, 2922–2926, 2852–2854, and 1452–1460 cm^−1^ assigned to stretching and bending vibrations of CH_3_ and CH_2_ groups in the fats and aliphatic amino acids [17,40].

The characteristic bands of the CH_2_ groups at 2922, 2852, and 1460 cm^−1^ were found to be more pronounced for adenocarcinomatous polyps due to the contribution of fats. The band or shoulder near 1740 cm^−1^ was assigned to the C=O stretching vibration in the fats. IR bands at 1396–1400 cm^−1^ were assigned to symmetric stretching of COO^−^ groups in the Asp and Glu residues of the proteins [40]. The band at 1236–1238 cm^−1^ originated from amide III vibrations in the proteins, =CH bending vibration in the unsaturated fatty acids, and antisymmetric stretching vibrations of PO_2_^−^ in the phospholipids [39,41]. The region of 930–1170 cm^−1^ had several overlapping bands assigned, mainly to CO and CC stretching vibrations in carbohydrates (glycogen and glycosaminoglycans) and lipids; the band at 1080–1084 cm^−1^ also contributed to symmetric stretching vibrations of PO_2_^−^ in the phospholipids and nucleic acids [36,41].

The FTIR difference spectra between both types of polyps and normal colon tissue, assigned as “a–n” and “c–n”, respectively, and between cancerous and adenomatous polyps, “c–a”, are represented in Figure 1b. The first two curves demonstrated positive bands at 1686, 1520, 1435, 1375, 1300, 1250, 1221, 1043, and 966 cm^−1^ that are characteristic of colon polyps of both types. These bands originated mainly from proteins, nucleic acids, and polysaccharides [37]. The positive bands at 1612 cm^−1^ (COO^−^ stretching) and 1105 cm^−1^ (CO and CC stretching) were more pronounced for adenomatous polyps; both of these bands may contribute to glycosaminoglycans. In contrast, the positive bands at 2918, 2850, 1734, and 1468 cm^−1^ indicated the prevalence of lipids in cancerous polyps. The last curve, “c–a”, showed several positive bands at 2918, 2850, 1736, and 1468 cm^−1^ (CH_2_ and C=O groups in lipids) and negative bands at 1612, 1498, 1396, and 1078 cm^−1^ (phenyl, carboxylate, and phosphate groups). The negative band at 1498 cm^−1^ with a shoulder near 1514 cm^−1^ could be assigned to in-plane C=C-H bending and C=C stretching vibrations of the phenyl ring in the aromatic amino acids Phe and Tyr, respectively [40,42,43,44].

#### 3.1.2. FT Raman Spectra (500–3100 cm^−1^)

The most intense band in the FT Raman spectra of both normal colon tissues and the colorectal polyps was found at ~2933 cm^−1^ (Figure 1c). This band corresponded mainly to the symmetric stretching vibration of the methyl groups in the aliphatic residues; the corresponding band of bending vibration was found at ~1448 cm^−1^. The shoulders at 2882, 2850, and 1460–1463 cm^−1^, assigned to the vibrations of the CH_2_ groups, were pronounced for the adenocarcinomatous polyps, which indicate an increased amount of lipids [45]. The bands at 3057–3061, and 1658 cm^−1^ originated mainly from amide vibrations in the proteins. Several bands of unsaturated compounds at 3008–3015, 1500–1590, and 1267 cm^−1^ were more pronounced for colorectal polyps than for normal colon tissue. Two bands of the polyps at 1609 and 1406 cm^−1^ arose from stretching vibrations of the COO^−^ groups. For all of the samples, the narrow band near 1004 cm^−1^ corresponded to the ring vibration of Phe in proteins. The bands in the region of 1120–1174 cm^−1^ mainly originated from CO and CC stretching vibrations of polysaccharides, and the bands in the region of 1077–1097 cm^−1^ originated from phosphate vibrations in the phospholipids and nucleic acids.

The FT Raman difference spectra between the diagnostic groups of colon tissues are shown in Figure 1d. The “a–n” and “c–n” curves demonstrated two positive regions of 1532–1685 cm^−1^ and 1250–1334 cm^−1^, which had contribution mainly from the vibrations of the peptide groups and aromatic amino acid side chains in the proteins. These areas may indicate an increased protein content in adenomatous polyps compared with the control; however, for adenocarcinomas, it is difficult to assess these differences due to the small number of samples and the high heterogeneity of this group. These areas may indicate an increased protein content in adenomatous polyps compared with the control; however, for adenocarcinomas, it is difficult to assess these differences due to the small number of samples and the high heterogeneity of this group. In all of these curves, the bands of CH_2_ vibrations found at 2877–2891, 2840–2852, and 1465–1470 cm^−1^ indicated that the lipid contribution was maximal for cancerous polyps and minimal for adenomatous polyps [46], while normal colon tissues demonstrated an intermediate contribution. Curve “c–a” showed several negative bands at 1648–1685 and 1230–1329 cm^−1^ associated mainly with the amide I and amide III vibrations of the proteins, respectively. The next positive region of 809–1083 cm^−1^ with the main maximum at 919 cm^−1^ contributes to the vibrations of lipids, polysaccharides and the phosphate groups [46]. These negative and positive parts of the curve correspond to the assumption about the possible predominance of proteins in adenomatous polyps and lipids and polysaccharides in adenocarcinomatous polyps. However, it is difficult to assert this with certainty because of the large standard deviation of the adenocarcinoma spectra.

#### 3.1.3. Dispersion Raman Spectra (450–3100 cm^−1^)

In the region of CH stretching vibrations (2800–3100 cm^−1^), the average dispersion Raman spectra of the normal colon tissue and two types of colorectal polyps (Figure 1e) have the most intense band assigned to the symmetric stretching of methyl groups at 2930–2932 cm^−1^ [45]. Several bands at 2889, 1675, 1589, 1409, 1330, 1271, 1144, 1057, and 941 cm^−1^ were pronounced in the spectrum of the normal colon tissue. In contrast, bands near 1340, 1308, 1208, 1174, 1153, 1123, 1088, 934, 873, 825, and 722 cm^−1^ were found in the spectra of the colon polyps. These spectral differences can be explained by expressive changes in the composition and structure of the biochemical tissue components, mainly proteins and lipids, and, to a lesser extent, polysaccharides and nucleic acids. The bands at 1656–1659 and 1259–1271 cm^−1^ arose from various vibrations of proteins and unsaturated lipids, and the bands at ~1448 and 1330–1340 cm^−1^ were assigned mainly to CH_2_, CH_3_, and CCH bending vibrations of aliphatic amino acids and lipids [45]. Bands at 1579–1589, 1205–1228, 1028–1033, 1002, 850–855, 825–828, and 739–746 cm^−1^ contribute from aromatic amino acids in proteins. Bands in the region of 1174–1102 cm^−1^ originated mainly from COC, CO, and CC vibrations in polysaccharides and lipids, and the band at 1083–1089 cm^−1^ arose from symmetric stretching vibrations of PO_2_^−^ groups in the phospholipids and nucleic acids.

The difference dispersion Raman spectra of “a–n” and “c–n” demonstrated positive difference bands at 2993–2995, 1655, 1520, 1369–1373, 1298–1301, 1205, 1125, 929, 845, 826, 780, 756, 718, and 666 cm^−1^ that are characteristic of colon polyps (Figure 1f). These bands contribute proteins, lipids, and unsaturated compounds (see Table S1). The band at 1084–1087 cm^−1^ was assigned to the vibration of phosphate groups, and the positive difference band near 845 cm^−1^ contributed to the C1αH bending vibration in glycogen. In contrast, the negative difference bands at 2866, 1677, 1588, 1491, 1410, 1351, 1329, 1272, and 1239 cm^−1^ were characteristic of normal colon tissues. The positive band at 2993–2995 cm^−1^ and the negative band at 2866 cm^−1^ (C-H stretching region) demonstrated the difference between normal colon tissues and polyps in the contributions of various alkyl moieties. The difference spectrum “c–a” had positive features at 2876, 2856, 1443, and ~1300 cm^−1^ originating from various vibrations of CH_2_ groups prevailing in lipids. The negative regions of 1590–1661, 974–1256, and 885–933 cm^−1^ mainly arose from protein vibrations. Two negative bands at 1077 and 717 cm^−1^ also contributed to the vibrations of phospholipids and nucleic acids, and the positive band at 847 cm^−1^ contributed to the C1αH bending of glycogen.

### 3.2. Discrimination of Normal Colon Tissues, and Adenomatous and Adenocarcinomatous Polyps

**Figure 2:**
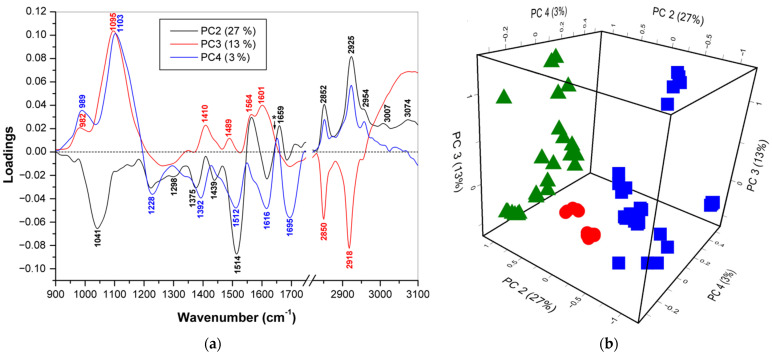

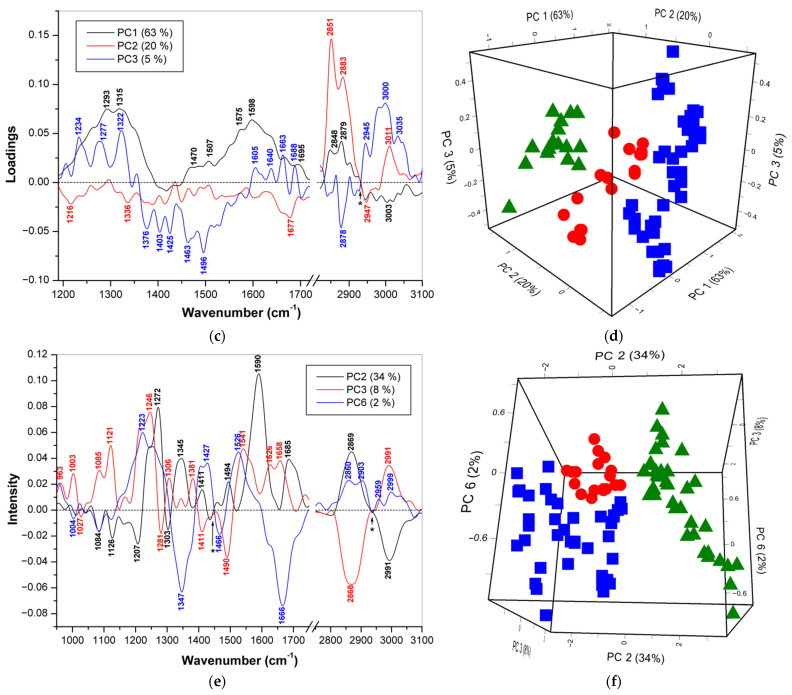
The loadings plots (left panels) and 3D component score plots (right panels) for three PCs of FTIR (**a**,**b**), FT Raman (**c**,**d**), and dispersion Raman (**e**,**f**) spectra of normal colon tissues (green triangles), adenomatous (blue squares), and adenocarcinomatous (red rings) colon polyps.

A principal component analysis (PCA) was conducted on the FTIR, FT Raman, and dispersion Raman spectra of the normal colon tissues (*n* = 10) and colon polyps (*n* = 10), the latter of which included adenomatous (*n* = 7) and adenocarcinomatous (*n* = 3) polyps measured at six independent locations for each sample. In each case, the combinations of two spectral regions of low and high wavenumbers were chosen for effective discrimination. For each vibrational spectroscopic method, the first six main components (PCs) were examined. Three of the six that showed significant differences between the three groups of tissue samples were chosen as the main discriminants. Although PC1 covers the largest variation in the dataset, specifically 53% for FTIR, 63% for FT Raman, and 42% for dispersion Raman, it sometimes does not show effective separation of the mentioned groups because of the strong differences among the individual cancerous polyps. So, for example, in the case of FTIR spectra, PC1 separated the first case of adenocarcinoma not only from the rest of the malignant polyps, but also from all other samples, and only PC2 separated the adenomatous polyps from the normal colon tissue, and the adenocarcinoma cases were located between two mentioned groups (Figure S3). Such discrimination could be explained by the higher content of lipids in adenocarcinomas, especially in the first case with greater invasiveness and a wide area of lesions, which was reflected in the spectra. Conversely, the adenoma samples showed less intense lipid bands than the normal tissue. The use of higher PCs made it possible to take into account the contribution of other biochemical components of colon tissues. To improve discrimination, higher PCs were used instead for the FTIR and dispersion Raman spectra. The loading plots of these sets of PCs are shown in Figure 2a,c,e. Three-dimensional component score plots (Figure 2b,d,f) were constructed involving the appropriate combination of PCs, i.e., PC2 (27%) vs. PC3 (13%) vs. PC4 (3%) for FTIR, PC1 (63%) vs. PC2 (20%) vs. PC3 (5%) for FT Raman, and PC2 vs. PC3 vs. PC6 for the dispersion Raman. These combinations proved to be the most suitable for distinguishing between normal colon tissue and both types of colon polyps.

#### 3.2.1. PCA/FTIR (900–1750 cm^−1^ and 2830–3100 cm^−1^)

According to the 3D score plot for FTIR, the adenomatous polyps had a negative PC2 score, while all of the other samples had a positive PC2 score, with the cancerous polyps located between the clusters of normal colon tissue and adenomatous polyps (Figure 2b).

The PC3 score, in turn, further separated the adenocarcinomatous polyps from the normal colon tissue cluster; the former had PC3 values lower than −0.35, and the latter had values higher than −0.35. Finally, the PC4 score focused the cancerous polyps around zero and thus also separated them from other samples with negative or positive values. The combination of these three PCs makes it possible to distinguish between all three groups of samples. The loading curve of PC2 has two positive bands at 2925 and 2852 cm^−1^, indicating the contribution of lipids in normal colon tissue and cancerous polyps, while the negative bands at 1514 and 1041 cm^−1^ assigned to C=C stretching and =CH bending vibrations in aromatic amino acids, respectively, could indicate higher amounts of proteins in adenomatous polyps (Figure 2a). The latter band also contributes to CO and CC vibrations in carbohydrates. The two lipid bands corresponding to those described above for PC2 were negative for PC3 and positive for PC4, which means that for these two components, the lipids remained key compounds for further discrimination. In addition, the strong positive PC3 and PC4 bands at 1095–1103 cm^−1^ indicated the contribution of phosphate groups in the phospholipids and nucleic acids, and two positive PC3 bands at 1601 and 1410 cm^−1^, as well as the corresponding negative PC4 bands at 1616 and 1392 cm^−1^, arose from carboxylate groups in the proteins (Asp and Glu). Therefore, IR bands of all of the mentioned compounds may contribute to PCA discrimination of colon tissues and colon polyps. However, the numerous bands of these biomolecules overlap significantly, making it difficult to determine which component is responsible for the spectroscopic difference at specific wavenumbers.

#### 3.2.2. PCA/FT Raman (1190–1750 cm^−1^ and 2820–3100 cm^−1^)

In the case of FT-Raman, none of the PCs used were able to completely separate the malignant polyps from the other two clusters, although PC1 clearly distinguished the normal colon tissues from the adenomatous polyps, the latter having higher PC1 values than the former. The border between these two clusters corresponded to a PC1 value of −0.5. In addition, the adenomatous polyps were separated by PC1 from the cancerous polyps that were on either side of the former. Additionally, the combination of the two subsequent components of PC2 and PC3 made it possible to completely separate the adenocarcinomatous polyps from the normal colon tissues. As a result, the use of all three mentioned PCs led to a complete separation of clusters of normal tissue and both types of polyps (Figure 2d). The loading curve of PC1 had three regions of positive bands at 1200–1400, 1450–1700, and 2830–2930 cm^−1^ and one less pronounced negative region at 2930–3100 cm^−1^ (Figure 2c). The PC2 curve had intense positive bands of lipids at 3011, 2851, and 2883 cm^−1^ corresponding to weaker positive PC1 bands at 2848 and 2879 cm^−1^, so positive values of PC2 indicated samples with high amounts of lipids. In contrast, negative PC2 bands at 1677, 1336, and 1216 cm^−1^ were associated with protein vibrations. Finally, the positive regions of PC3 at 2945–3035 cm^−1^ (=CH stretching), 1605–1688 cm^−1^ (amide I and C=C stretching), and 1234–1322 cm^−1^ (amide III and =CH bending) arose mainly from unsaturated compounds and proteins, while the negative PC3 bands at 2870, 2840, 1463, 1376, and 1299 cm^−1^ were assigned to the vibrations of the CH_2_ and CH_3_ groups. As a result, the discrimination was based on differences in the relative contributions of the proteins and lipids, and the ratio between the saturated and unsaturated groups in these biomolecules was also significant.

#### 3.2.3. PCA/Dispersion Raman (950–1750 cm^−1^ and 2750–3100 cm^−1^)

In the case of the dispersion Raman spectra, PC2 was able to discriminate the normal colon tissues from colon polyps. Indeed, as seen from the 3D component score graph (Figure 2f), the normal colon tissue cluster was located in the positive PC2 region, while all colon polyps were in the negative region. In addition, the use of a combination of PC3 and PC6 was effective for distinguishing between adenomatous and malignant polyps. Two intense positive bands of the PC2 loading curve at 1590 and 1272 cm^−1^ were assigned to the C=C stretching and =CH bending vibrations of the aromatic compounds, respectively (Figure 2e). These bands probably originated from the hem moiety of blood hemoglobin [47,48] or rather tissue hemoproteins, including myoglobin and cytochromes [49,50]. These bands were enhanced due to the pre-resonance effect, and their positions were displaced due to the denaturation of hemoproteins and changes in the hem environment. The bands at 2991 and 2868 cm^−1^, which were attributed to various C-H stretching vibrations, had opposite signs on the PC2 and PC3 loading curves. Positive bands of PC3 at 1246 and 1085 cm^−1^, in turn, indicated the contribution of phosphate groups. Finally, three negative bands of PC6 at 1666, 1347, and 1004 cm^−1^ mainly arose from proteins, partially collagen. Consequently, the biochemical background of this discrimination was rather complex and included the contribution of various biomolecules.

#### 3.2.4. LDA Classification of Colon Tissues/Polyps Using Vibrational Spectroscopic Data

From the above results, it is evident that the PCA discrimination of normal colon tissue and polyps based on FTIR, FT Raman, or dispersion Raman spectra is not satisfactory for the diagnosis of colorectal carcinoma. Indeed, in the component score plots the clusters of normal colon tissues and adenomatous and adenomatous polyps are very close to one another, and therefore it should be difficult to assign a borderline sample to a specific cluster.

**Figure 3:**
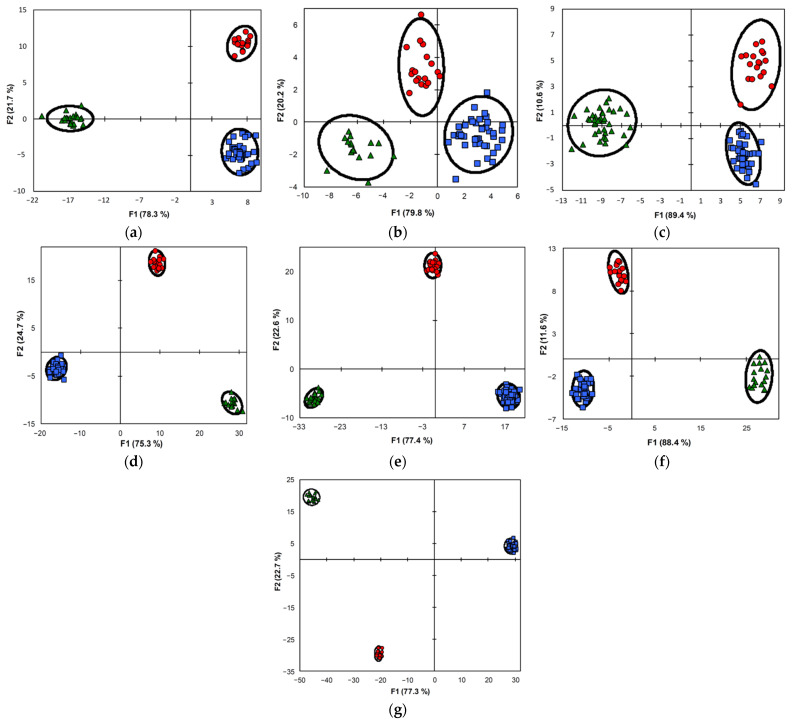
The LDA discrimination of normal colon tissues (green triangles), adenomatous (blue squares) and adenocarcinomatous (red rings) colon polyps on the factor score plots for FTIR (**a**), FT Raman (**b**), dispersion Raman (**c**), FTIR + FT Raman (**d**), FTIR + dispersion Raman (**e**), FT Raman + dispersion Raman (**f**), and FTIR + FT Raman + dispersion Raman (**g**) datasets with the confidential ellipses.

**Figure 4:**
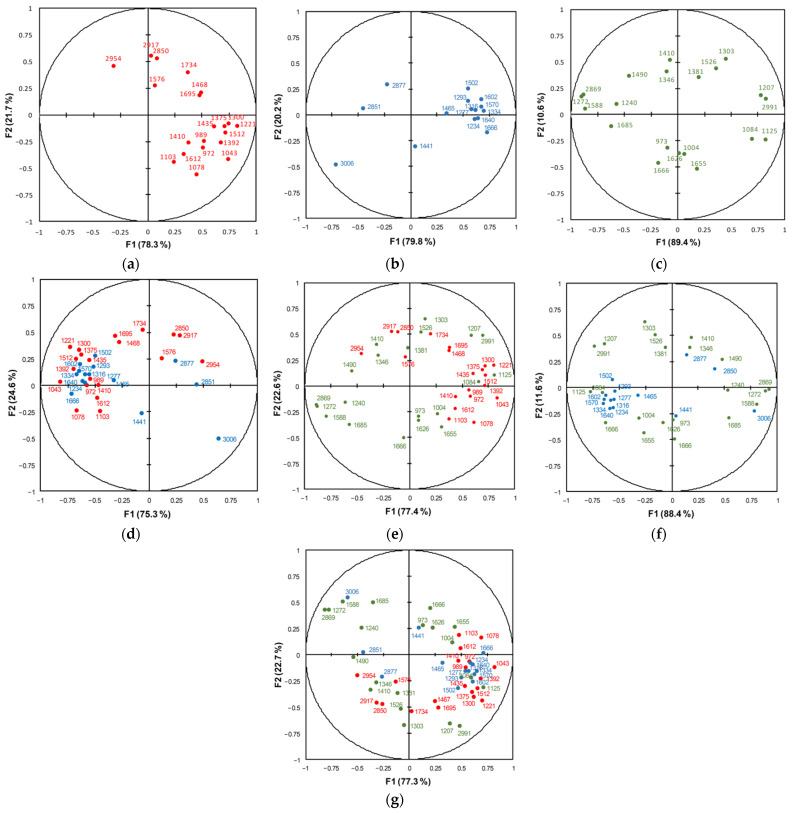
Variables/factor correlation plots for FTIR ((**a**), red), FT Raman ((**b**), blue), dispersion Raman (**c**, green), FTIR + FT Raman ((**d**), red and blue), FTIR + dispersion Raman ((**e**), red and green), FT Raman + dispersion Raman ((**f**), blue and green), and FTIR + FT Raman + dispersion Raman ((**g**), all colors) datasets.

**Figure 5:**
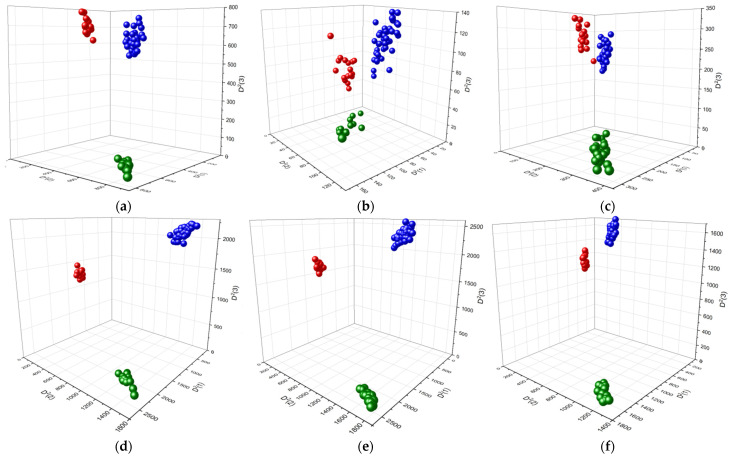

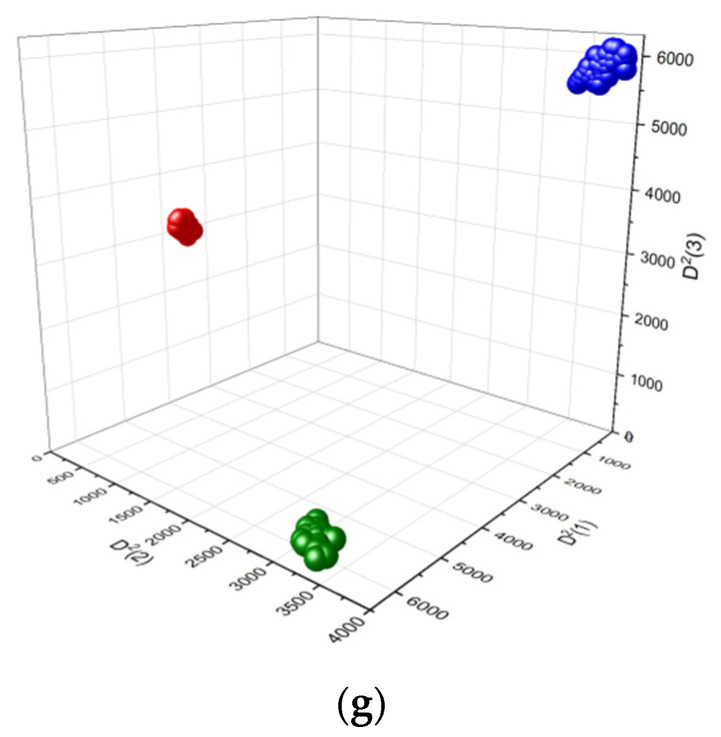
The squared Mahalanobis distances calculated for FTIR (**a**), FT Raman (**b**), dispersion Raman (**c**), FTIR + FT Raman (**d**), FTIR + dispersion Raman (**e**), FT Raman + dispersion Raman (**f**), and FTIR + FT Raman + dispersion Raman (**g**) datasets demonstrating discrimination of normal colon tissues (green triangles), adenomatous (blue squares), and adenocarcinomatous (red rings) colon polyps by LDA.

To improve discrimination, 15–20 wavenumber values per vibrational spectroscopic method were chosen for the LDA classification (Table 1). The selection of these three sets of variables was made based on a comparison of the difference spectra (Figure 1b,d,f) and the extremes of individual PCs (Figure 2a,c,e). The chosen variables corresponded to specific tissue components, mainly lipids and proteins, but polysaccharides, nucleic acids, and other metabolites may contribute to some of them (see Table S1 for the interpretation). The results of the LDA discrimination of the samples using individual sets or their combination are represented in the factor score plots, variables/factor correlation plots, and the 3D plots of the squared Mahalanobis distances (Figure 3, Figure 4 and Figure 5). In all of the cases, the samples were successfully separated into three clusters of normal colon tissue, adenomatous and adenocarcinomatous polyps.

The FTIR method demonstrated discrimination between the clusters of normal colon tissue and colon polyps of both types according to F1, and values of F2 additionally separated the clusters of adenomatous polyps from the cluster of cancerous polyps (Figure 3a). A similar but not as effective discrimination was obtained by the dispersion Raman, where one point of malignant polyps was located in proximity to the cluster of benign polyps (Figure 3c). In contrast, in the case of FT Raman, all three clusters were separated by F1, and F2 additionally separated the cluster of adenocarcinomatous polyps from the other two (Figure 3b). However, in this case, the clusters were rather diffuse and situated close to one another, especially those of the two types of colon polyps. The combination of any two methods led to a significant improvement of discrimination in comparison with the individual methods (Figure 3d–f). It is also evident that LDA for the combined model, including all three methods (55 variables), represented the best discrimination. All three clusters were very compact and located in different quadrants, and were well separated from one another, and the points of each cluster were densely packed into narrow areas (Figure 3g).

The correlation between the chosen variables corresponding to specific wavenumbers is represented in variable/factor correlation plots (Figure 4). The FTIR variables were separated by F2 into two groups, one positive and one negative (Figure 4a). The former group (seven variables) mainly corresponded to vibrations of lipids (2917, 2850, 1734, and 1468 cm^−1^) and could be nucleic acids or unfolded proteins (2954, 1695, and 1576 cm^−1^); these bands could be markers of a malignancy for colon polyps. The latter group (13 variables) mainly corresponded to specific vibrations of proteins (including collagens), nucleic acids, and polysaccharides pronounced for adenomatous polyps. Most FT Raman variables were associated with proteins and were located in the region of positive F1, while only three variables of lipid vibrations (3006, 2877, and 2851 cm^−1^) were found in the negative region of F1 (Figure 4b). In contrast, the dispersion Raman variables (Figure 4c) were arranged more or less diffusely, but it was possible to distinguish the clusters corresponding to the vibrations of proteins (1666, 1655, 1626, 1004, and 973 cm^−1^), unsaturated compounds (2869, 1685, 1490, 1272, and 1240 cm^−1^), and polysaccharides (1125 and 1084 cm^−1^). In the case of the combined sets of variables (Figure 4d–g), the variables/factor plot demonstrates the correlation between variables from different methods. For example, in the case of a combined FTIR + FT Raman dataset, the variables corresponding to the stretching vibrations of CH in aliphatic groups at 2954–2850 cm^−1^ are located in quadrant I (Figure 4d). In the case of triple combination, the main group associated with proteins and polysaccharides was located in the region of positive F1, while the lipid bands were found in quadrant III (negative F1 and F2) and six vibrations associated with unsaturated compounds in quadrant IV (Figure 4g). These three regions coincided with clusters of adenomatous polyps, adenocarcinomatous polyps, and normal colon tissue.

The LDA discrimination of the three diagnostic groups mentioned above using individual sets of variables or their combination is represented in 3D plots as squared Mahalanobis distances (Figure 5), describing both the distance between groups and the distance of individual group members (samples) from the group center [51]. The values of these distances for individual datasets are summarized in Table S2. It is evident that all methods discriminated these three groups more or less effectively, however, using the combined pair datasets (Figure 5d–f) led to better results compared to the corresponding individual datasets (Figure 5a–c). The best discrimination was obtained using the triple combined dataset which significantly improved the individual vibrational spectroscopic methods (Figure 5g). In this case, three compact groups were located at a considerable distance from one another. To evaluate the separation of diagnostic groups, the distances between them were calculated based on the squared Mahalanobis distances from the LDA of all the datasets (Table 2). The distances obtained by LDA of the triple combined dataset (~4800–8130) were an order of magnitude longer than those obtained by the LDA of the individual spectroscopic datasets that increased in the raw FTIR (~330–1050)—dispersion Raman (~88–360)—FT Raman (~64–330). A significant increase in the distances was also achieved for all pair of combined datasets (~517–3243). Among the diagnostic groups, the longest distances were observed between normal colon tissues and one of the groups of colorectal polyps, adenomatous (FT Raman and all combined) or adenocarcinomatous (FTIR and dispersion Raman) polyps. For all but the triple combined spectroscopic datasets, the smallest distance was between the benign and malignant colon polyps. For the combined datasets, the differences between these distances were not as significant for the separation, since the diagnostic groups were compact and sufficiently distant from one another.

## 4. Discussion

Significant spectral differences between normal colon tissues and adenomatous and adenocarcinomatous colon polyps described in the previous chapter were explained by the difference in the contributions of the main tissue constituents, including lipids, nucleic acids, proteins, and polysaccharides. The characteristic bands of these compounds demonstrated more or less sensitivity to the neoplastic transformation of the colon tissue.

Currently, many malignant neoplasms, including colorectal carcinoma, are accompanied by significant changes in lipid metabolism [52]. Consequently, some lipids may be potential biomarkers of the stage and risk of this disease. Indeed, the current investigation confirmed that, based on the average spectra of colorectal polyps, vibrational bands of lipids at ~2918, 2877–2891, 2840–2852, ~1734, 1465–1470, and ~1300 cm^−1^ could be assigned as spectral markers of a cancerous transformation. In addition, the phosphate vibration bands in the regions of 1080–1089 and 1234–1238 cm^−1^, which mainly arose from phospholipids and nucleic acids, as well as the bands of unsaturated fatty acids at 3008–3015, 2889, 1653–1659, and 1259–1271 cm^−1^, also contributed to the discrimination between normal colon tissues and colorectal polyps. However, this contribution was not as pronounced because of the overlapping protein and/or carbohydrate bands. The intense lipid bands found in the spectra of malignant polyps could be associated with an increase in the tissue content of these compounds compared with the normal colon tissue, as previously shown in some reports [53,54]. Partially, an increase in the total level of phosphatidylcholine was observed in the later stages of colorectal carcinoma [55].

LDA analysis of the current study has shown that vibrational bands of nucleic acid at about 2950–2960, 1574–1576, 1483–1484, 1310–1372, and 966 cm^−1^ may contribute to the identification of colon polyps. The increase in the DNA content, together with other biomarkers of high or abnormal DNA activity, have been successfully used to identify more aggressive colorectal adenomas with an increased potential for malignant transformation [56]. The DNA index, shape, and density of the nucleus have proven to be valuable indicators for distinguishing between normal colon mucosa, adenoma, and adenocarcinoma [57], and thus the DNA contribution to vibration spectra of the colon tissues and colon polyps, especially the bands associated with the vibrations of purine nucleotide bases (A, G), could be significant for the early diagnosis of CRC. 

In the current work, it was determined that the characteristic vibrational bands of proteins, in particular collagens, are very promising for distinguishing normal colon tissues from benign and malignant colorectal polyps. In general, it was determined that bands associated with vibrations of peptide groups and amino acid side chains, including those specific for collagens (Pro, Hyp), were more pronounced for colon polyps than for normal colon tissue. The differences in collagen marker bands point to the changes in the amount, structure, and conformation of these proteins. Changes in collagen density and alignment have been detected in high-grade dysplasia polyps and malignant specimens [58]. The diagnostic factor may not be the content of collagen in the cancer tissue, but the ratio between the individual structural forms (subtypes) of this protein that play specific roles in carcinogenesis. For example, collagen I inhibits the differentiation of human colorectal carcinoma cells and promotes their stem cell-like phenotype [59], and changes in the collagen I expression could be an early event in colorectal carcinoma informative for prognosis [60]. In contrast, the expression of collagen XVII correlates with the invasion and metastasis of colorectal cancer [61]. In addition, the collagen IV content was significantly correlated with the stage and histological grade of colorectal carcinoma [62].

The polysaccharides of the intercellular matrix, known as glycosaminoglycans (GAGs), also contribute to the vibrational spectra of colon tissues and colon polyps, especially in the regions of 1660–1400, 1260–1210, 1200–950, and 860–810 cm^−1^, corresponding to stretching/bending vibrations of CONH/COO^−^, S=O, CO/CC, and HCS bonds in these polysaccharides, respectively [63,64,65,66]. GAGs undergo significant structural alterations in cancer, including changes in their hydrodynamic size and sulfation patterns [67]. The amount of individual polysaccharides and more complex proteoglycans can also be changed. For example, the heparin sulfate and chondroitin sulfate levels were significantly increased in colon carcinomas, while the level of collagen-associated proteoglycan decorin decreased in some cases [68]. Typically, the content of chondroitin sulfates in cancerous tissue is significantly higher than that in normal tissue. In colon cancer, the level of chondroitin/dermatan C-4 sulfate increased in terms of normal adjacent and tumor tissues by approximately 1.5 times compared with the healthy control tissue, while the content of chondroitin C-6 sulfate increased by 2.5 times in tumor tissue compared with normal adjacent tissue, and non-sulfated chondroitin was found only in the tumor tissue [69]. In addition to sulfated GAGs, hyaluronic acid is also a promising prognostic marker for colorectal carcinoma [70,71]. Despite the high variability of this polysaccharide in the cytosol attributed to the biological heterogeneity of colorectal cancer, high cytosolic levels have been associated with a poor prognosis.

Glycogen, an energy storage polymer of glucose, is also known as a metabolic marker of colorectal carcinoma. Carcinogenesis often leads to an unusual increase in glycogen storage because it affects the cellular catabolism involving glucose [72]. In the current work, despite overlapping by other vibration modes, the CO and CC stretching vibrations of this polysaccharide at 950–1150 cm^−1^ may contribute to a spectroscopic distinction between normal colon tissues, and adenomatous and adenocarcinomatous colon polyps. Glycogen levels were measured early in normal and cancerous human colorectal tissues [73]. The highest glycogen levels were found in adenocarcinoma, which were lower in the normal colon tissue and the lowest in the areas proximal to the tumor. The accumulation of glycogen varied with the type and stage of adenoma or adenocarcinoma. In adenocarcinomas, the level of glycogen depends on the phase of cell proliferation, since malignant tissues are able to supply themselves with glycogen and take it from the surrounding tissues as an energy source for the biosynthesis of DNA and proteins. 

Summarizing what has been discussed above regarding the biopolymeric components of the colon tissues, it should be noted that the contribution of these macromolecular substances to the vibrational spectra of tissues is not always clear due to the strong overlap of the bands. Therefore, it is often difficult to determine which of these components is a chemical marker of pathological changes. Despite some uncertainty in the interpretation of the number of bands in the FTIR and Raman spectra, the application of multivariate statistical methods, namely the combination of PCA and LDA, in the evaluation of vibrational spectroscopic data, made it possible to successfully distinguish ex vivo benign and malignant colon polyps from one another and from normal colon tissue. Such a combination of vibrational spectroscopy and multivariate statistics can serve as an additional tool for the early diagnosis of colorectal carcinoma.

## 5. Conclusions

Average FTIR, FT Raman (λ_ex_ 1064 nm), and dispersion Raman (λ_ex_ 785 nm) spectra of normal colon tissue, and benign and malignant colon polyps obtained from surged or endoscopic patients demonstrated evident spectral differences in terms of the intensities and positions of characteristic bands assigned to vibrations of main tissue constituents, i.e., proteins, lipids, polysaccharides, etc. A suitable statistical model allowed us to discriminate normal, abnormal, and cancerous colon tissue samples based on a multivariate analysis of the vibrational spectroscopic data. LDA classification was especially successful in the case of combined set of 55 variables from FTIR, FT Raman, and dispersion Raman spectra. This model can be proposed for ex vivo colorectal cancer diagnostics in combination with colonoscopic extraction of colon polyps for further testing. The pilot study presented here is a precursor for the further evaluation of diagnostic potential for simultaneous in vivo application of endoscopic Raman probes.

## Figures and Tables

**Table 1:** Variables (cm^−1^) of the FTIR, FT Raman and dispersion Raman spectra used for the discrimination of normal colon tissues, and benign and malignant colon polyps.

Method	Variables: Wavenumber (cm^−1^)
FTIR	972, 989, 1043, 1078, 1103, 1221, 1300, 1375, 1392, 1410, 1435, 1468, 1512, 1576, 1612, 1695, 1734, 2850, 2917, 2954
FT Raman	1234, 1277, 1293, 1316, 1334, 1441, 1465, 1502, 1570, 1602, 1640, 1666, 2851, 3006
Dispersion Raman	973, 1004, 1084, 1125, 1207, 1240, 1272, 1303, 1346, 1381, 1410, 1490, 1526, 1588, 1626, 1655, 1666, 1685, 2869, 2991

**Table 2:** Distances between the diagnostic groups *d_ij_* determined by LDA using seven datasets: normal colon tissues “n”, adenomatous “a”, and adenocarcinomatous “c” colon polyps.

Dataset	Distances between the Diagnostic Groups (Mean ± SD)		
	n ↔ a	n ↔ c	a ↔ c
FTIR	959.2 ± 43.5	1048.2 ± 39.4	329.0 ± 25.8
FT Raman	329.0 ± 17.4	83.6 ± 16.4	63.8 ± 16.4
Dispersion Raman	314.9 ± 31.9	360.5 ± 33.5	88.3 ± 24.6
FTIR + FT Raman	2859.5 ± 69.8	1944.4 ± 65.8	1793.5 ± 65.0
FTIR + Dispersion Raman	3243.1 ± 79.0	2548 ± 70.2	1648.0 ± 68.6
FT Raman + Disp. Raman	2243.3 ± 72.0	1965.1 ± 73.4	516.9 ± 61.5
FTIR + FT Raman + Disp. Raman	8127.4 ± 146.6	4798.5 ± 118.3	5718.0 ± 102.8

## Data Availability

Not applicable.

## Supplementary Materials

The following are available online at https://www.mdpi.com/article/10.3390/diagnostics11112048/s1, Figure S1a–c: FTIR (a), FT Raman (b) and dispersion Raman (c) spectra of the model standard compounds, Figure S2. Average FTIR ATR spectra and 2nd derivations of these spectra of the normal colon tissues, adenomatous and adenocarcinomatous colon polyps, Figure S3. The component score plot PC1 versus PC2 for FTIR spectra of the normal colon tissues, adenomatous and adenocarcinomatous (red rings) colorectal polyps. Table S1: Wavenumbers (in cm^−1^) and assignments of vibration bands for the average spectra of normal colon tissue “n”, adenomatous “a” and adenocarcinomatous “c” colon polyps, Table S2: Squared Mahalanobis distances for normal colon tissues “n”, adenomatous “a” and adenocarcinomatous “c” colon polyps determined by LDA using four spectral datasets.

## Appendix A

The squared Mahalanobis distances *D*^2^(1), *D*^2^(2) and *D*^2^(3) (mean ± SD) represented in Table S2 were calculated for each diagnostic group, i.e., normal colon tissues “n”, adenomatous colon polyps “a” and adenocarcinomatous colon polyps “c”, from the corresponding values obtained for each measurement site by the LDA classification using the XLSTAT software (Addinsoft, France). The distances between the three diagnostic groups *d_ij_* (*i* and *j* are the diagnostic groups) represented in Table 2 were calculated from the squared Mahalanobis distances of the corresponding groups according to the equation:

(A1) $$d_{ij}\;=\sqrt{{\left(D^{2}{\left(1\right)}_{i}-D^{2}{\left(1\right)}_{j}\right)}^{2}+{\left(D^{2}{\left(2\right)}_{i}-D^{2}{\left(2\right)}_{j}\right)}^{2}+{\left(D^{2}{\left(3\right)}_{i}-D^{2}{\left(3\right)}_{j}\right)}^{2}}$$
