# Supplementary material for: Ex Vivo Vibration Spectroscopic Analysis of Colorectal Polyps for the Early Diagnosis of Colorectal Carcinoma
Source: Diagnostics (Basel). 2021 Nov 4;11(11):2048. doi: 10.3390/diagnostics11112048 (PMC8621094; doi:10.3390/diagnostics11112048)
Supplement: Supplementary file 1 [file diagnostics-11-02048-s001.zip › diagnostics-1428214-supplementary.pdf]

Supplementary Materials

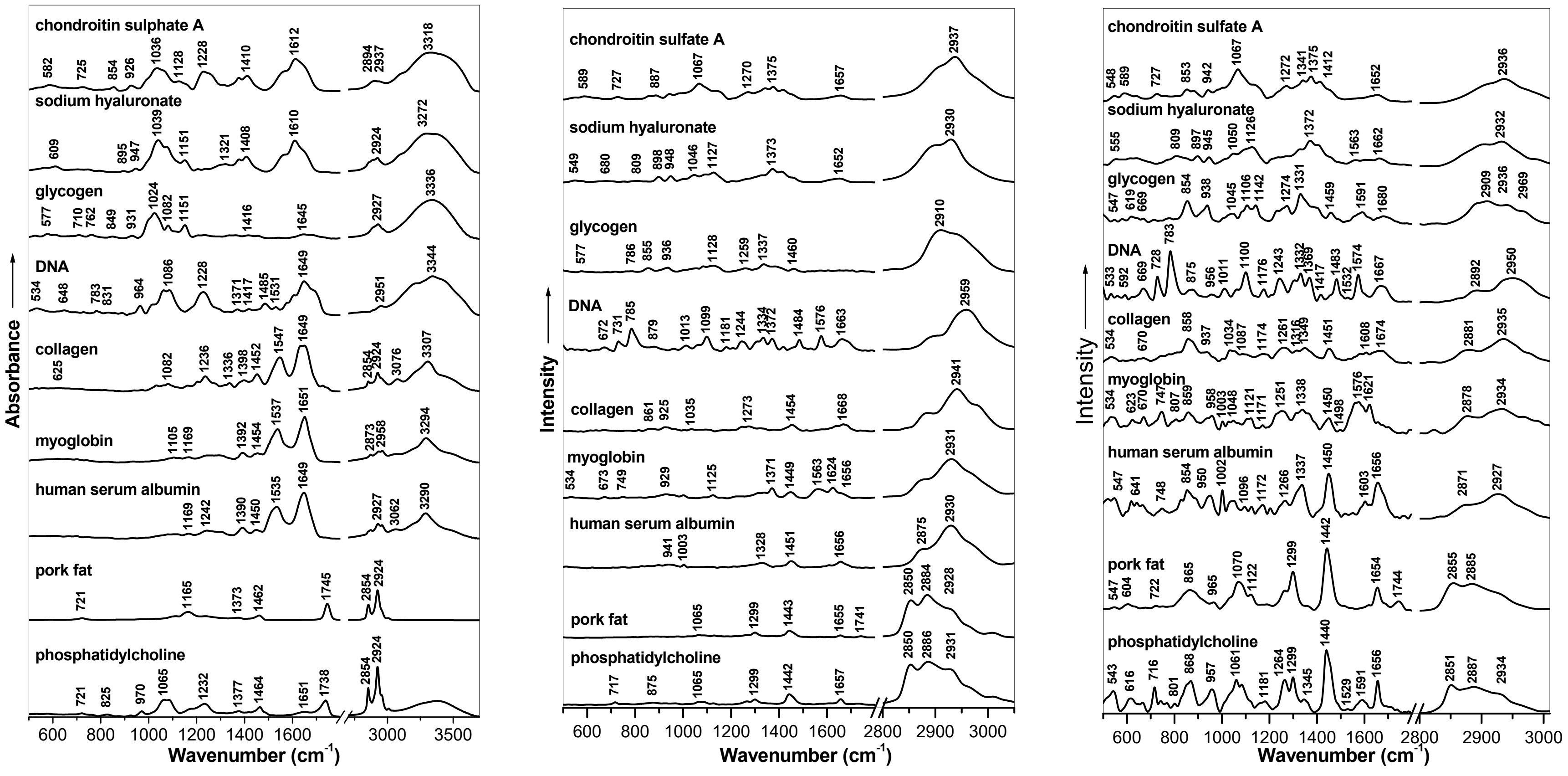

(**a**) (**b**) (**c**)

Figure S1. FTIR (**a**), FT Raman (**b**), and dispersion Raman (**c**) spectra of the model standard compounds.

Table S1. Wavenumbers (in cm^{-1}) and assignments of vibration bands for the average spectra of the normal colon tissue "n", adenomatous "a" and adenocarcinomatous "c" colon polyps.

**FTIR**			**FT Raman**			**Dispersion Raman**			**Vibrational mode**	**Compounds**[1]
**n**	**a**	**c**	**n**	**a**	**c**	**n**	**a**	**c**		
3290	3282	3286							amide A	prot.
3080	3086	3082	3057	3059	3061				amide B	prot.
			3008sh	3014sh	3015sh				ν(=CH)	lipids (UFA)
2958	2956	2954	2972sh	2963sh	2960sh	2968sh	2976sh	2979sh	$\nu_{as}(CH_3)$	lipids, prot. (aliph.)
			2937sh	2933	2935	2933	2931	2934	$\nu_{s}(CH_3)$	prot. (aliph.), lipids
2926	2926	2918							$\nu_{as}(CH_2)$	lipids
						2889			$\nu_{as}(CH_2)$	lipids (UFA)
			2872sh	2876sh	2882	2870sh	2873sh	2874sh	$\nu_{as}(CH_2)$	lipids
2872	2873	2872sh							$\nu_{s}(CH_3)$	prot. (aliph.), lipids
2854	2854	2852	2850sh	2850sh	2850sh	2849sh	2849sh	2850sh	$\nu_{s}(CH_2)$	lipids
1740	1734sh	1734sh	1741	1740	1742				ν(C=O)	lipids
1649	1647	1649	1658	1658	1658	1659	1657	1656	amide I, ν(C=C)	prot., lipids (UFA)
			1619sh			1611sh		1616	ν(C=C)	prot. (Tyr, Phe)
1632sh	1633sh	1633sh	1605sh	1609	1609		1600	1600	ν(C=C), $\nu_{as}(COO^-)$	prot. (Glu, Asp)
						1589			ν(C=C)	lipids (UFA)
1570sh		1575sh	1583		1589	1574sh	1579	1572	ν(C=C), ν(C=N)	prot. (Phe, Trp), NA (A)
1547	1539	1539	1560	1560		1553sh	1549sh	1545	amide II, ν(C=C)	proteins

			1538sh	1528	1528	1534sh		1522	ν(C=C)	proteins
1513sh			1518sh			1514sh	1517sh		ν(C=C)	proteins
1496sh			1497sh		1506	1489sh		1497sh	ν(C=C), ring	NA (G)
1468sh	1468sh	1460	1460sh	1463sh	1462sh	1462sh		1463sh	δ(CH_2)	lipids
1454	1452	1452sh	1447	1449	1447	1448	1447	1447	δ_{as}(CH_3)	prot., lipids
1400	1396	1394			1406	1409		1410sh	ν_s(COO^-)	prot. (Glu, Asp)
						1383	1388	1387	δ_s(CH_3)	prot. (aliph.)
1344			1341		1338	1330	1339	1340	δ(CCH)	prot. (aliph.), glyc.
1313			1322sh		1316				δ(CCH), amide III	prot. (aliph.)
	1309	1306	1302	1305	1300	1303sh	1302sh	1308	τ(CH_2), amide III	lipids, proteins
1284			1271	1263sh	1267	1271	1259	1261	amide III, δ(=CH)	prot. (α-helix), lipids (UFA)
1238	1234	1236		1238sh	1242sh	1243sh			amide III, ν_{as}(PO_2^-)	prot. (α-helix), NA (OPO)
1205sh			1210	1206sh	1202sh		1205sh	1208	ν(CPh)	prot. (Trp, Phe)
1167	1171sh	1170	1174	1167	1178	1170	1174	1173	ν(CO), ν(CC), δ(CH)	lipids, glyc., prot. (Tyr)
	1157sh	1153sh	1158		1156	1144	1154	1152	ν(CO), ν(CC), δ(CH)	lipids, glyc., prot. (Tyr)
1120sh	1120sh	1120sh	1126	1127	1128	1122sh	1123	1124	ν(CO), ν(CC), δ(CH)	lipids, glyc., prot.
1084	1080	1080	1084	1097	1081	1083sh	1088	1089	ν_s(PO_2^-), ν(CO), ν(CC)	NA (OPO), PL
			1065	1077	1072	1057	1064sh	1059sh	ν(CO), ν(CC), δ(CH)	lipids, glyc., prot.
1051sh	1047sh	1043sh	1044	1046sh	1046			1044sh	ν(CO), ν(CC), δ(CH)	lipids, glyc., prot.
1032sh			1032sh	1030sh	1031	1029	1033	1028sh	δ(CH)	proteins (Phe)
			1004	1003	1003	1002	1002	1002	ring breath.	proteins (Phe)

974	974	970		946		959	965	955	ν(CO), ring	NA (OPO, dRib)
933			936	937	931	941	935	934	ν(NCαC), ν(CC)	proteins (α-helix), lipids
			888	882	898	884sh	873	872	ring breath.	proteins (Tyr, Pro)
			857	859	855	855	853	850	δ(C1αH)	glyc.
			826sh	828	810	826sh	828	825	ring breathing	prot. (Tyr, Pro, Hyp)
			809sh	798	802	805sh	802sh	804sh		
777sh			781	783		771sh	779	779	ν(OPO)	PL
742sh			760	754	758	739	744	746	ring breath.	prot.(Trp)
721sh			718	718	721		721	723	ring breath., τ(CC)	NA (A), lipids
698	698	698	697	681	688		693sh	694sh	ring breath., τ(CC)	NA (G)
661	660		672		665	667	666	667	ring breath., ν(CS)	NA (T), prot. (Cys)
			644	642	643	642sh	644	641	τ(CC)	lipids, prot. (Tyr)
619	617	623	621	619	622	619sh	622	619	τ(CC)	lipids, prot. (Phe)
		548	554	554	556	549sh	542	544	τ(CC), ν(SS)	prot. (Pro, SS bridges)
			502			505sh	504sh	511	ν(SS)	prot. (SS bridges)

[1] alyph., aliphatic amino acids; glyc., glycogen; NA, nucleic acids; PL, phospholipids; prot., proteins; UFA, unsaturated fatty acids; sh, shoulder.

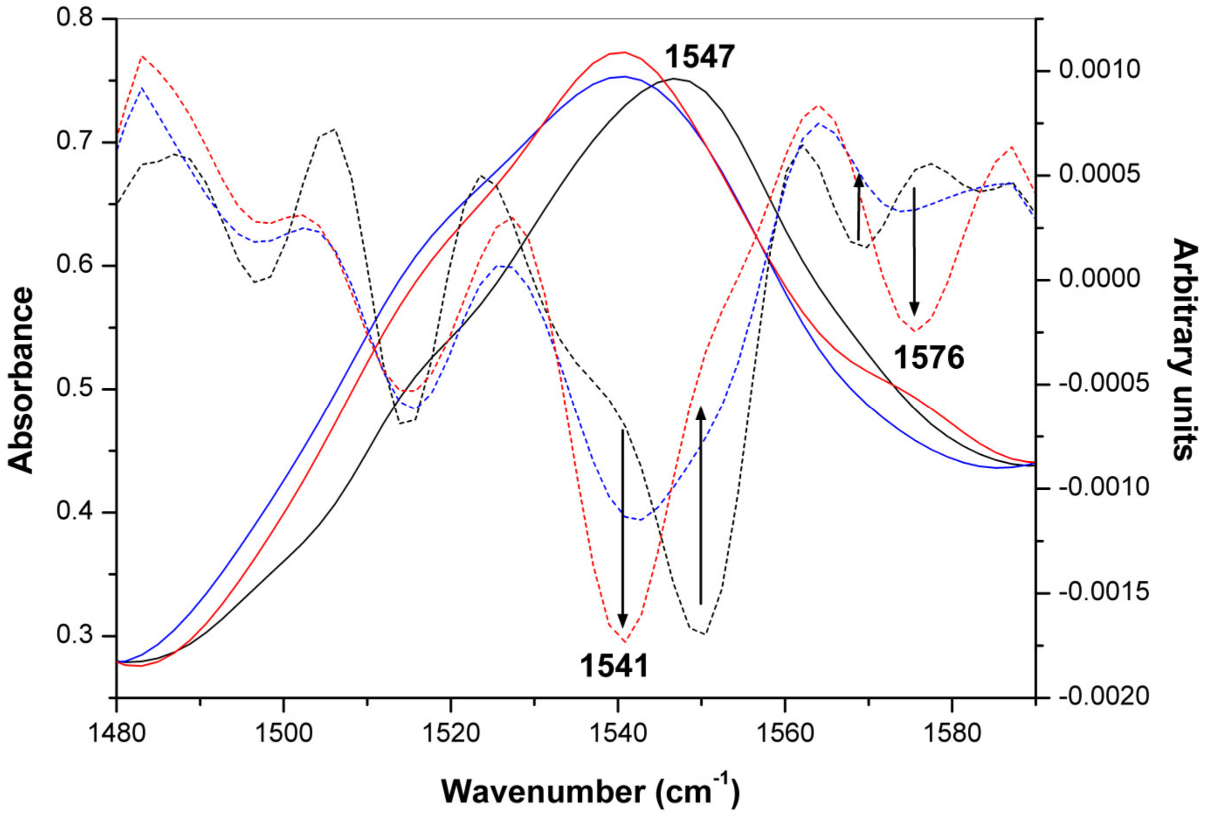

Figure S2. Average FTIR ATR spectra (solid lines) and 2nd derivations of these spectra (dash lines) of the normal colon tissues (black), adenomatous (blue) and adenocarcinomatous (red) colon polyps in the region of 1480–1590 cm^{-1}.

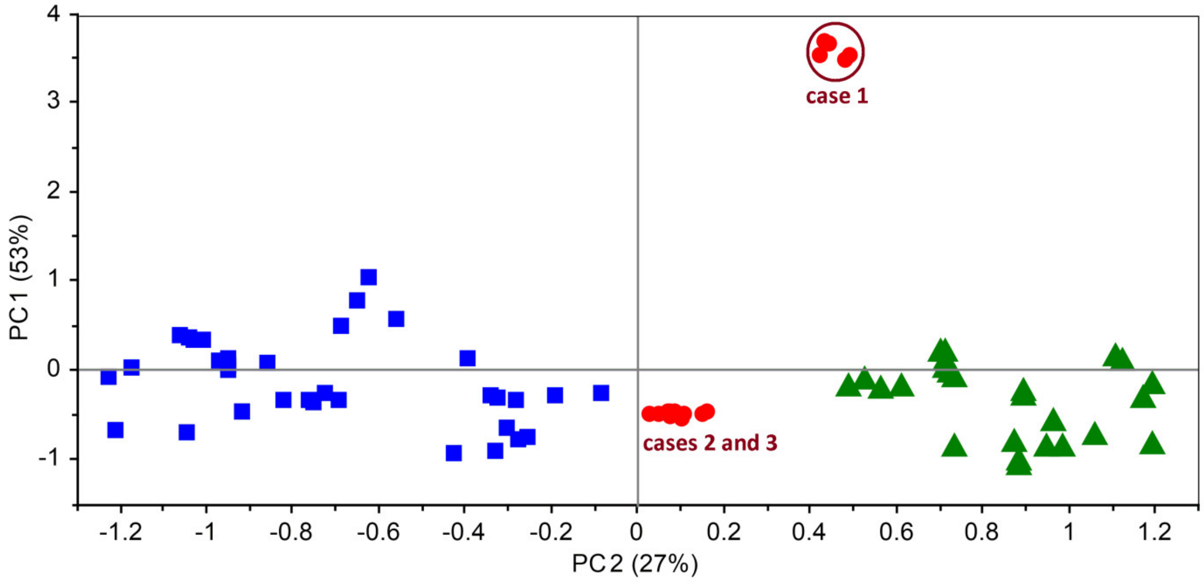

Figure S3. The component score plot PC1 versus PC2 for FTIR spectra of the normal colon tissues (green triangles), adenomatous (blue squares) and adenocarcinomatous (red rings) colorectal polyps.

Table S2. Squared Mahalanobis distances for the normal colon tissues "n", adenomatous "a" and adenocarcinomatous "c" colon polyps determined by LDA using four spectral datasets.

**Dataset**	**Diagnostic groups**	**Squared Mahalanobis distances D^2 (mean ± SD)**		
		**$D^2(1)$**	**$D^2(2)$**	**$D^2(3)$**
FTIR	n	614.3 ± 41.4	714.6 ± 41.9	22.0 ± 7.4
	a	23.2 ± 8.1	248.7 ± 27.2	616.6 ± 37.4
	c	237.0 ± 13.1	14.9 ± 2.6	705.2 ± 32.3
FT Raman	n	96.6 ± 15.3	60.1 ± 11.6	11.2 ± 6.1
	a	16.0 ± 4.6	51.9 ± 10.2	101.4 ± 16.3
	c	49.8 ± 11.0	13.9 ± 4.2	62.8 ± 13.6
Disp. Raman	n	233.2 ± 28.0	287.5 ± 32.5	22.7 ± 8.5
	a	19.5 ± 5.5	70.0 ± 11.0	230.3 ± 18.2
	c	70.6 ± 15.8	23.5 ± 7.0	285.3 ± 26.6
FTIR	n	2058.9 ± 65.3	1255.6 ± 58.1	37.9 ± 8.6
+ FT Raman	a	35.3 ± 7.4	1165.7 ± 58.3	2056.2 ± 72.0
	c	1156.1 ± 48.2	25.6 ± 6.7	1243.3 ± 60.5
FTIR	n	2306.2 ± 71.1	1631.3 ± 64.2	43.6 ± 10.8
+ Disp. Raman	a	36.2 ± 10.6	1099.9 ± 60.0	2298.0 ± 83.2
	c	1101.0 ± 54.3	37.3 ± 8.0	1624.9 ± 59.3
FT Raman	n	1505.2 ± 80.2	1122.3 ± 70.0	35.1 ± 9.3
+ Disp. Raman	a	32.7 ± 6.4	279.5 ± 25.0	1502.8 ± 57.5
	c	281.2 ± 23.1	34.4 ± 7.7	1121.6 ± 55.6
FTIR	n	5791.2 ± 160.6	3079.2 ± 102.7	56.5 ± 7.0
+ FT Raman	a	50.3 ± 6.2	3610 ± 91.6	5785.0 ± 128.6
+ Disp. Raman	c	3611.2 ± 65.0	51.2 ± 5.2	3073.8 ± 89.1
